# An Intra-Oral Optical Sensor for the Real-Time Identification and Assessment of Wine Intake

**DOI:** 10.3390/s19214719

**Published:** 2019-10-30

**Authors:** Paul Faragó, Ramona Gălătuș, Sorin Hintea, Adina Bianca Boșca, Claudia Nicoleta Feurdean, Aranka Ilea

**Affiliations:** 1 Bases of Electronics Department, Electronics, Faculty of Telecommunications and Information Technology, Technical University of Cluj-Napoca, 400027 Cluj-Napoca, Romania; Ramona.Galatus@bel.utcluj.ro (R.G.); Sorin.HINTEA@bel.utcluj.ro (S.H.); 2 Department of Histology, Faculty of Medicine, University of Medicine and Pharmacy “Iuliu Hatieganu” Cluj-Napoca, 400012 Cluj-Napoca, Romania; bianca.bosca@umfcluj.ro; 3 Department of Oral Rehabilitation, Oral Health and Dental Office Management, Faculty of Dentistry, University of Medicine and Pharmacy “Iuliu Hațieganu” Cluj-Napoca, 400012 Cluj-Napoca, Romania; feurdeanclaudia@gmail.com (C.N.F.); aranka.ilea@umfcluj.ro (A.I.)

**Keywords:** optical fiber sensors, wearable sensors, intra-oral sensors, optical coupler, side-emitting fiber, wine spectroscopy, wine color analysis

## Abstract

Saliva has gained considerable attention as a diagnostics alternative to blood analyses. A wide spectrum of salivary compounds is correlated to blood concentrations of biomarkers, providing informative and discriminative data regarding the state of health. Intra-oral detection and assessment of food and beverage intake can be correlated and provides valuable information to forecast the formation and modification of salivary biomarkers. In this context, the present work proposes a novel intra-oral optical fiber sensor, developed around an optical coupler topology, and exemplified on the detection and assessment of wine intake, which is accounted for example for the formation of N^ε^-carboxymethyllysine Advanced Glycation End-products. A laboratory proof of concept validates the proposed solution on four white and four red wine samples. The novel optical sensor geometry shows good spectral properties, accounting for selectivity with respect to grape-based soft drinks. This enables intra-oral detection and objective quality assessment of wine. Moreover, its implementation exploits the advantages of fiber-optics sensing and facilitates integration into a mouthguard, holding considerable potential for real-time biomedical applications to investigate Advanced Glycation End-products in the saliva and their connection with consumption of wine, for the evaluation of risk factors in diet-related diseases.

## 1. Introduction

Clinical diagnosis is commonly based on invasive procedures for the determination of disease-signaling blood biomarkers. Although blood biomarkers are considered to be the most relevant in the diagnostics procedure [1], the research community targets to investigate the collection of biomarkers from alternative body fluids: sweat, saliva, tears, etc. [2,3,4,5].

Human saliva is indeed an attractive oral fluid, and its preference over other body fluids mainly consists of the ease of collection and relative simplicity of analysis, while exhibiting a reduced risk of cross-contamination and infection spread [6]. Indeed, a wide spectrum of compounds present in the salivary fluid has been proven as highly informative and discriminatory, and could be considered as targeted analytes for intra-oral sensors in order to investigate the oral and general health status of an individual [7]. At the present time, several electrochemical sensors have been developed for the analysis of various salivary components such as: glucose, urea, cytokines, mucins, and Advanced Glycation End-products. Moreover, it was determined that salivary compounds are well correlated to the blood concentrations of numerous analytes [8]. Thus, saliva analysis enables a painless diagnostic alternative to accurately reflect the healthy vs. diseased state conditions in humans, particularly useful for people with nervousness concerning the collection of blood samples or for those who require frequent clinical monitoring with multiple sampling in a relatively narrow time interval, e.g., every hour, multiple times per day, etc.

Point-of-care biosensensors are developed to help in the early diagnosis, periodic monitoring, and treatment of disease. Wearable sensors have recently received considerable interest for real-time monitoring of different parameters, specific for the wearer’s health, in a wide range of biomedical point-of-care biosensors [5], sport [9], diet-related diseases [10], and military scenarios [11]. Most of the activity on wearable sensors has focused either on the non-invasive monitoring of vital signs via electrophysiological signals, as, for example, electrocardiography, electromyography, photopletismography, pulse oximetry, etc. [12,13,14], or on chemical sensing for chemical biomarker detection in body fluids [15,16,17,18].

The intra-oral concept has gained considerable interest as a wearable biosensing platform with real-time monitoring. For exemplifications, denture-based sensors have been reported for pH and temperature monitoring in [19]. Another example consists of a mouthguard biosensor for continuous salivary-based monitoring of metabolites, as reported in [20].

Integration of electrochemical sensor into the oral cavity is strongly limited by the necessity of a power supply, e.g., external supply, battery or energy harvesting, and corresponding electronics. As such, another direction of research targets in vivo monitoring with battery-less operation of the wearable sensor [21]. A solution for bacteria monitoring in the saliva, reported in [22], resembles a dental tattoo consisting of a resistor, inductor and capacitor (RLC) resonant circuit which has the resistance value, and, consequently, the resonant frequency, varied in the presence of the salivary compounds of interest. Supplying and reading the sensor are performed over the resonant coil, thus eliminating the need for onboard power and external connectivity.

Alternatively to electrophysiological monitoring and electrochemical sensing, optical sensing, in the shape of wearable and autonomous equipment, is enabled by 25 years of tremendous growth and development in the field of fiber optics. Fiber optic sensors offer a wide range of advantages over traditional sensing systems: small size for integration, ease of manufacturing and adaptation for patient-dedicated components, electrical passivity and immunity to electromagnetic interferences, electrical isolation, safety for interaction with the human body as well as environmentally friendly in the sense that fiber optics do not contaminate their surroundings [23,24]. From the signal point of view, fiber optic sensors exhibit a wide dynamic range and high sensitivity, thus having the ability to monitor a wide array of physical and chemical parameters.

Various technologies, including infrared spectroscopy, fluorescence spectroscopy, Raman spectroscopy, liquid chromatography–mass spectrometry (LC-MS), and gas chromatography–mass spectrometry (GC-MS), were proven capable of sensitive detection of different sample compounds, but require expensive equipment and complicated operation for home-based care applications. The optical fiber-based platform offers a suitable, sensitive alternative for point-of-care monitoring of different chemical parameters [25].

Advanced Glycation End-products (AGEs), familiarly known as Maillard products, have recently gained interest as novel biomarkers in the evaluation of risk factors in diet-related diseases. AGEs are inherently formed in foods, and are hypothesized for their endocrine disrupting properties considered as a significant concern for public health [26]. Endocrine disruptors (ED) are either natural or synthetic chemicals (such as pesticides, metals, additives or contaminants in food, and personal care products), which, at certain dosage, interfere with hormones of the living system. Thus, they have been suspected to be associated with health defects such as abnormal growth patterns and neurodevelopmental delays in children, as well as changes in the immune function, altered reproductive function in males and females, and increased incidence of breast cancer [27]. It is estimated that about one thousand out of one hundred thousand manufactured chemicals have endocrine disrupting properties [28]. As such, a new field of research aims towards the development of new salivary biosensor platforms for AGE identification [29,30,31].

Human exposure to EDs occurs via ingestion of food, water, alcohol, and dust, as well as inhalation of gases and particles in the air and through the skin. For instance, MG (methylglyoxal), GO (glyoxal), and 3-DG (3-deoxyglucosone) derived AGEs are highly formed during manufacturing of bakery products, carbonated soft drinks sweetened with high fructose corn syrup and fermented foods such as yoghurt, wine, or beer. Thus, the assessment of food and beverage intake provides results very well correlated to the formation of AGEs [32]. For a concrete exemplification, the intake of wine is correlated to the formation of N^ε^-carboxymethyllysine (CML) and accounts for the intake of 11.20–32.80 AGEs kU/100 mL (estimated for Pinot Noir and Pinot Gris, respectively) [33], and is far more elevated in the cases of chronic alcohol misuse [34]. While salivary biomarker sensing is difficult to be performed with purely optical means, an alternative approach assumes to rather perform identification and assessment of wine in order to forecast the formation and/or modification of targeted biomarkers.

In this paper, we propose a novel intra-oral fiber-optics-based sensor geometry, aiming for the detection and assessment of wine intake. We have previously demonstrated fiber-optics-based salivary optical sensing in [35]. On the other hand, we have illustrated that intra-oral label-free sensing with a D-shaped side-polished optical fiber sensor is limited. Nevertheless, the implementation of intra-oral sensors with plastic optical fibers (POF) accounts for a series of advantages such as flexibility and ease of integration into an intra-oral device. Moreover, fiber-optics sensors can be straightforwardly extended with the deposition of a labeling layer, as well as a noble metal layer for surface plasmon resonance (SPR) [36].

The proposed sensor geometry drops the traditional transmission configuration [37] in favor of an optical coupler topology. The proof-of-concept intra-oral optical sensor is developed around a POF deployed in a T-optical coupler topology, having optical radiation applied laterally into a D-shaped side-polished optical fiber. Unlike the previously reported salivary sensor in [35], which is implemented around a fluorescent optical fiber and performs analyte detection via the modification of the fluorescence spectrum, the intra-oral sensor proposed in this work performs analyte identification via its spectral signature. Depending on the spectral analysis specifications for analyte identification, this can be performed on either the radiation spectrum, the transmission spectrum, or the absorption spectrum, respectively. In the present exemplification, which targets wine detection and assessment, sensing will be performed by investigating the absorption spectrum, and only the visible range will be considered. Two types of radiation sources are investigated: a broad-band white LED source to implement point sensing, and a side-emitting fiber to extend the sensing area. To replicate intra-oral sensing conditions, the proposed optical sensor is integrated into a mouthguard support. Tests are then carried out in a laboratory environment to validate the proposed sensor laboratory proof of concept.

## 2. Materials and Methods

### 2.1. Point Intra-Oral Sensor

**Figure 1:**
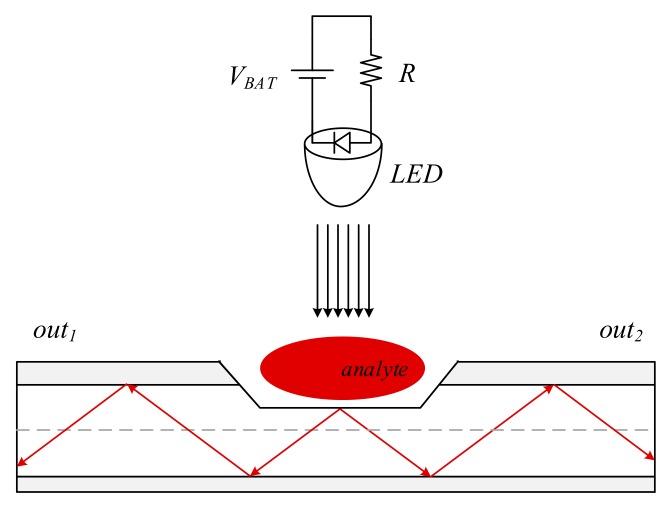
Implementation concept of the proposed intra-oral optical sensor, consisting of an LED source and a D-shaped side-polished optical fiber deployed in a vertical coupler topology, implementing point sensing.

The proposed intra-oral optical sensor implementation concept is illustrated in the diagram from Figure 1, resembling point sensing. The proposed optical sensor is developed around a D-shaped side-polished POF. Rather than having the fiber propagate optical radiation applied axially on one end, which is the case for side-polished evanescent-wave optical fiber sensors [36,37,38,39], the proposed solution assumes having optical radiation applied vertically into the polished fiber surface, implementing an optical coupler topology.

Operation of the proposed intra-oral sensor is described as follows. A white LED source (supplied from battery voltage *V_BAT_* over bias resistance *R*) applies wide-band radiation onto the polished surface of the optical fiber, i.e., the sensing area. This radiation is coupled into the optical fiber and is transmitted to the fiber receiving end. An analyte, present on the sensing area of the fiber, filters the incident radiation and consequently modifies the parameters of the propagated radiation. A spectrometer, deployed on the fiber receiving end, can then be employed to assess the spectrum of the propagated radiation, enabling analyte identification based on its spectral signature.

As illustrated, the proposed intra-oral sensor concept implements vertical coupling of the optical radiation into the fiber, resembling the topology of a T coupler [40,41]. Accordingly, the T-coupler spectral attenuation measures, expressed in terms of insertion loss, coupling ratio, and excess loss, are considered for spectral characterization of the proposed intra-oral sensor. 

Insertion loss (*IL*) is expressed as

(1) $$IL_{i}\left(\lambda \right)=10\mathrm{lg}\frac{P_{in}\left(\lambda \right)}{P_{out,i}\left(\lambda \right)},$$

where *P_in_*(*λ*) is the power of the incident light source and *P_out,i_*(*λ*), *i* = {1, 2}, is the power measured at both optical fiber receiving ends. 

Coupling ratio (*CR*) is expressed as the difference between the insertion loss measurements in dB, at the two fiber ends, as

(2) $$CR\left(\lambda \right)=IL_{1}\left(\lambda \right)-IL_{2}\left(\lambda \right).$$

Excess loss (*EL*) is expressed as

(3) $$EL_{i}(\lambda )=10\mathrm{lg}\frac{P_{in}(\lambda )}{P_{out,1}\left(\lambda \right)-P_{out,2}\left(\lambda \right)}.$$

As expressed in Equations (1)–(3), the insertion loss, coupling loss, and excess loss are wavelength dependent. Thus, the spectral attenuation measures are employed to characterize the intra-oral sensor in the wavelength domain. 

The proposed intra-oral sensor exhibits several advantages in comparison to a classical evanescent-wave side-polished optical fiber sensor. On one hand, lateral application of the incident radiation through the polished fiber surface eliminates the limiting effects of sensing area length and polishing depth, which is the case with evanescent-wave optical sensors [38]. On the other hand, lateral illumination of the optical fiber accounts for increased flexibility and is more convenient for applying light into the fiber core, while relaxing the specifications for expensive coupling optics, which is the case for axial illumination [42,43,44]. In addition, the proposed sensor topology can be straightforwardly applied to extend the sensing area.

### 2.2. Extended Sensing Area Intra-Oral Sensor

**Figure 2:**
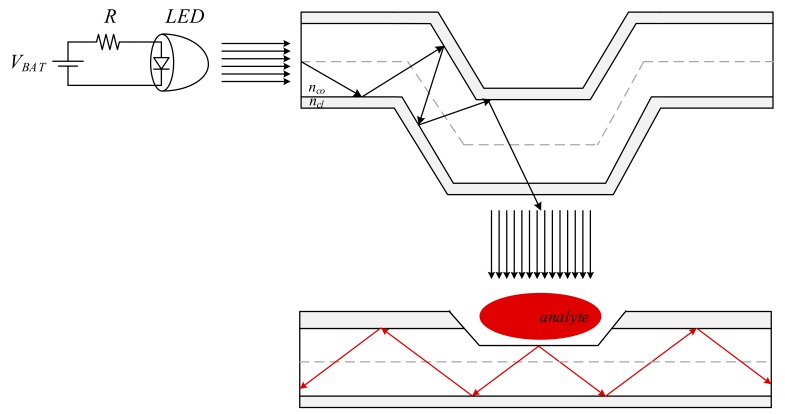
Implementation concept of the extended sensing area intra-oral sensor, consisting of an LED source, a side-emitting fiber, and an end-emitting fiber. Sensing is achieved by having the end-emitting fiber incident illumination applied via a side-emitting fiber, which distributes the incident LED source onto the entire sensing surface.

For the realization of extended area sensing, the sensing area on the optical fiber is extended by having polished a longer fiber section in contrast to the point sensor. Then, the incident radiation must also be distributed to feed light into the optical fiber along the entire sensing area. For this purpose, a side-emitting fiber was deployed as illustrated in the diagram of the extended sensing area intra-oral sensor from Figure 2.

As illustrated, the side-emitting fiber and the end-emitting optical fiber are deployed in parallel, in an emitter–receiver structure, implementing vertical coupling of the optical radiation [40]. The sensing procedure is the same as for the point sensor. The analyte present on the sensing area will filter the incident illumination, thus modifying the parameters of the propagated radiation. However, in contrast to the point sensor, the extended sensing area sensor enables intra-oral detection along the whole polished fiber length.

Some considerations regarding the deployment of the side-emitting fiber must be specified at this stage. Propagation of light in an optical fiber is performed via total internal reflection (TIR), provided that the core and the cladding refraction indices, *n_co_* and *n_cl_* respectively, follow

(4) $$n_{co}>n_{cl},$$

determining a critical angle *θ_c_* expressed by

(5) $$\theta_{c}=90^{{}^{\circ}}-\arcsin\left(\frac{n_{cl}}{n_{co}}\right)=\arccos\left(\frac{n_{cl}}{c_{co}}\right).$$

In contrast to the end-emitting optical fiber, the core and cladding refraction indices of side-emitting fibers are chosen such as to violate the TIR condition expressed in Equation (4) and have the refraction indices be approximately equal [45]. As a consequence, radiation applied axially into the side-emitting fiber fiber will gradually leave the fiber via diffraction during propagation. Typically, the side glow intensity *I_S_* decreases exponentially with length,

(6) $$I_{s}(d)={\left(4\pi \right)}^{-1}\cdot I_{in}\cdot (1-\exp(-kd)),$$

where *k* is the scattering coefficient, *I_in_* is the input source intensity, and *d* is the distance variable along the fiber length [46]. Considering, however, that the targeted fiber length for integration into the intra-oral device is of only a few centimeters, it is sensible to consider the side-emission intensity constant.

**Figure 3:**
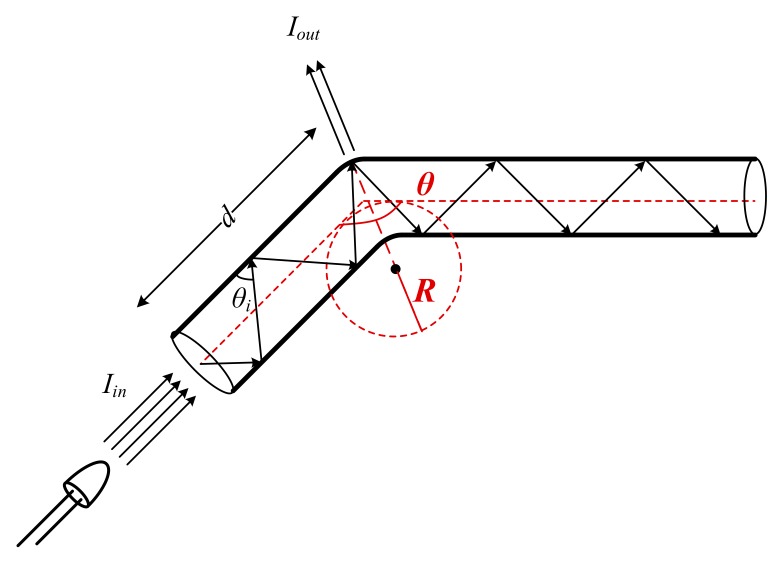
A macrobending of an optical fiber, with the illustration of the input source intensity *I_in_*, intensity of the light, which leaves the fiber through the bending *I_out_*, the propagation parameters: incidence angle *θ_i_*, and length variable *d*, and the bending parameters: bending radius *R* and bending angle *θ*.

With respect to integration into the intra-oral device, bending the side-emitting fiber to fit the curvature of the mouthguard produces a macrobending. By definition, a macrobending of the fiber is a curvature with a radius larger than the core diameter [47,48], as depicted in Figure 3.

The macrobending accounts for a geometric asymmetry in between the fiber core and cladding [45], which alters the incidence angle as follows. The macrobending produces a local change of the core refractive index profile expressed by [44,47,48,49]:

(7) $${n_{co}}^{\prime }\left(r,\theta \right)=\sqrt{n_{co}{}^{2}(r)+\frac{2n_{cl}{}^{2}}{R}r\cos\theta },$$

where *R* is the bending radius, *θ* is the bending angle, and *r* is the radial distance from the fiber axis. The critical bending radius *R_c_* is derived as

(8) $$R_{C}=\frac{2n_{cl}\lambda }{4\pi \sqrt{{\left(n_{co}-n_{cl}\right)}^{3}}}.$$

Accordingly, the core refractive index *n_co_’* can be decreased, as prescribed in Equation (7), by increasing the bending radius *R* above *R_c_*:

(9) $$R↑(>>R_{C})\Rightarrow n_{co}{}^{\prime }↓,$$

and increasing the bending angle *θ* around or above the critical angle *θ_c_*, yet below 90° [48]:

(10) $$\theta ↑(\geq \theta_{C})\Rightarrow n_{co}{}^{\prime }↓.$$

This violates the TIR condition in Equation (4) even further, translating to a larger amount of light leaving the fiber. This determines an increased side-glow intensity, while, on the other hand, it determines a propagation loss *a_MB_* expressed as

(11) $$a_{MB}(d,\lambda ,r,\theta )[dB]=10\mathrm{lg}\exp(2\cdot d\cdot \alpha_{MB}(\lambda ,n_{co}{}^{\prime }(r,\theta ))),$$

where *α_MB_(λ, n_co_’)* is the macrobending loss coefficient. The phenomenon of propagation loss due to fiber macrobending is undesirable for fiber optics data transmission. In this work, however, it contributes to the increase of the side-glow intensity of the side-emitting fiber, and, consequently, the optical sensor incident light source intensity.

As was the case with the point intra-oral sensor, the extended sensing area sensor is assimilated to an optical coupler, with the side-emitting fiber as the emitter and the end-emitting fiber as the receiver. The sensor is then characterized in the spectral domain as an optical coupler. However, considering the nature of the sensor, assimilation to an optical coupler assumes a continuum of emitters and receivers along the sensing area, making it impractical to assess individual loss components (for example, in terms of insertion or excess loss, respectively) as was the case with the point sensor.

On the other hand, the extended sensing area intra-oral sensor is also affected by additional sources of loss. The side-emitting fiber exhibits a wavelength dependency of the side glow, given by the scattering coefficient *k* in Equation (6), as well as by the macrobending parameters *R_c_* and *a_MB_* defined in Equations (8) and (11), respectively. The scattering coefficient of side-glow fibers exhibits an inverse proportionality vs. wavelength [50]. With respect to propagation, this accounts for a strong attenuation of short wavelengths (blue to green) for short distances and the transmission of long wavelengths (yellow to red) towards longer distances. This phenomenon was confirmed by our laboratory measurements. On the other hand, the bending parameters *R_c_* and *a_MB_* exhibit a direct proportionality vs. wavelength [51]. The scattering coefficient *k* and the macrobending parameters *R_c_* and *a_MB_* follow opposite wavelength dependency trends, with different magnitudes.

As such, an input/output spectral attenuation measure would be better suited to assess the wavelength dependency of the superimposed losses. For this purpose, we have considered the wavelength dependent coupling loss [40,52] as a spectral measure of the optical coupler for the characterization of the proposed extended sensing area intra-oral optical sensor. The wavelength dependent coupling loss (*CL*) is defined as an input/output ratio:

(12) $$CL\left(\lambda \right)=10\mathrm{lg}\frac{P_{in}\left(\lambda \right)}{P_{out}\left(\lambda \right)}$$

and resembles a superposition of individual loss components, rather than providing an individual estimate for each individual loss component which occurs during propagation and coupling, as was the case with the point sensor. Thus, the benefit of employing this measure is that, besides insertion loss and excess loss, the wavelength dependent coupling loss of the extended sensing area intra-oral sensor accounts for the wavelength dependency of the side-emitting fiber input/side-glow relationship, as well as the wavelength dependency of the bending loss.

### 2.3. Sensor Integration Into a Mouthguard

Two laboratory proofs of concept have been realized for the proposed intra-oral optical sensor: one for the point sensor and one for the extended sensing area sensor. The parameters of the plastic optical fibers are as follows: a 1 mm diameter end-emitting optical fiber with a 1.49 core refractive index and a 0.5 numerical aperture, and a 1 mm side-emitting fiber with a 1.525 core refractive index and a 0.6 numerical aperture.

**Figure 4:**
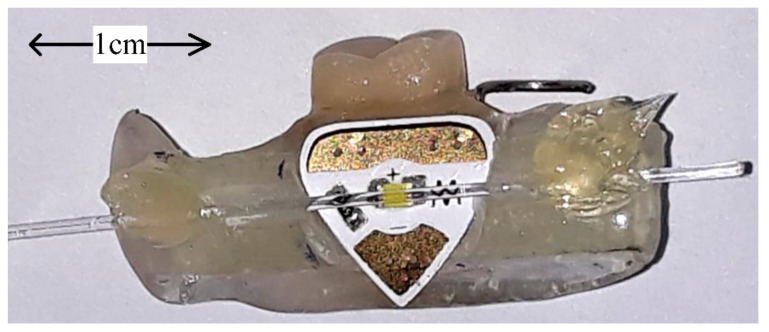
Practical realization of the point intra-oral sensor laboratory proof of concept, which has the LED source and the optical fiber from the sensor structure integrated into a lateral mouthguard.

The point intra-oral optical sensor consists of an LED source that applies light laterally into a side-polished optical fiber. A Chibitronix SMD form factor white LED (Chibitronics, Lewes, DE, USA), mounted on a flexible triangular sticker substrate alongside the bias resistance and pads for a 3V external power supply, was employed for this purpose. The D-shaped side-polishing of the end-emitting optical fiber was realized using the Industrial Fiber Optics IF CPK 2000 grit polishing paper (Industrial Fiber Optics, Inc., Tempe, AZ, USA). Polishing was realized by describing an 8-shaped motion with the bended fiber on the polishing film. The footprint of the LED and the fiber has been drilled into the mouthguard. The LED was deployed underneath the side-polished optical fiber, with a 0.5 mm spacing in between the LED and the fiber, such as to have the light applied over the polishing into the fiber core, as illustrated in Figure 4.

**Figure 5:**
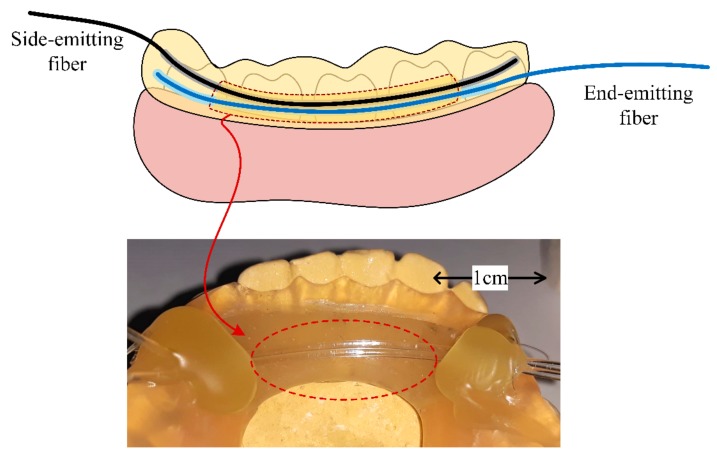
Practical realization of the extended sensing area intra-oral sensor laboratory proof of concept, which has the side emitting fiber and the end-emitting fiber deployed in parallel and integrated into a frontal mouthguard. The source, which feeds light axially into the side-emitting fiber, is external to the sensor.

The extended sensing area intra-oral optical sensor consists of a side-emitting fiber and an end-emitting optical fiber, deployed in parallel in a vertical coupler topology. Integration into the mouthguard follows the diagram illustrated in Figure 5 and is described as follows.

Two parallel canals were drilled into the mouthguard, such as to have the fibers deployed in parallel with a 0.5 mm spacing. This deployment is then parallel to the lingual slope and perpendicular to the dental arch. The advantage of this deployment is that the sensing surface is inherently exposed to the saliva.

Particular care was paid to having the curvature of the side-emitting fiber envisioned at this stage of the integration process. The side-emitting fiber and the side-polished end-emitting fiber are next fitted into the designated canals, such that the polished surface of the end-emitting optical fiber is oriented towards the curvature of the side-emitting fiber. To counter the inherent elasticity of the plastic fiber, a thin layer of acrylic resin was used to fix the fibers, thus preventing them to jump out from the designated canals. The sensing surface, however, was left exposed to the intra-oral environment in the shape of a window.

### 2.4. Spectral Analysis of Wine Color

**Figure 6:**
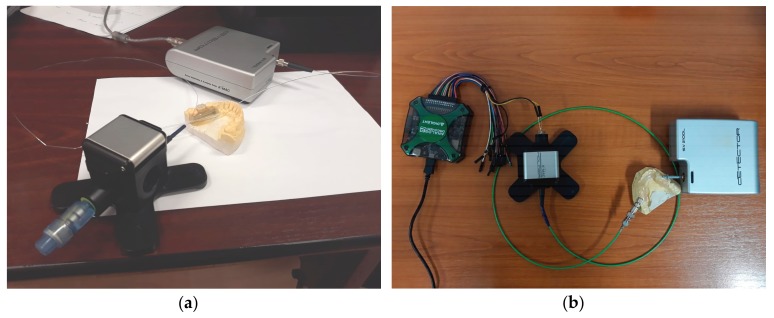
The intra-oral optical sensor test setup for wine color analysis: (**a**) point sensor test setup, and (**b**) extended sensing area sensor test setup.

Wine color analysis is performed in this work, with the proposed intra-oral optical sensor, by acquiring and assessing the wine absorption spectrum in the visible range, via the proposed intra-oral optical sensor. The measurement setup is depicted in Figure 6. A white LED applies wide-band radiation onto the analyte, either directly or via a side-emitting fiber, depending on whether the point sensor or the extended sensing area sensor is being tested. A KMAC SV2100 spectrometer (KMAC, Daejeon, Korea) is employed for the acquisition of the sensor output spectrum.

**Figure 7:**
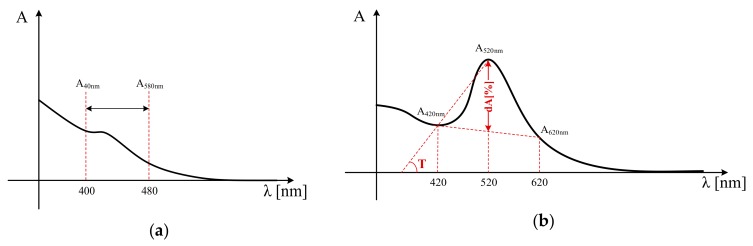
Qualitative plot of a typical absorption spectrum for: (**a**) white wines, and (**b**) red wines.

The typical absorption spectra of white and red wines, respectively, are illustrated in the qualitative plots from Figure 7. There are specific particularities in the shape of the wine absorption spectra that are exploited for wine color analysis. White wines exhibit a peak of the absorption spectra in the 400–480 nm wavelength range. Red wines, on the other hand, exhibit an absorption maximum at 520 nm, i.e., absorption of red—standing for the representative absorption of anthocyanins and their flavylium combinations, and a trough at 420 nm, i.e., absorption of yellow—standing for the absorption of tanins and flavonols [53].

Wine color is mainly defined in terms of intensity, chromaticity, and brightness [53,54,55,56]. Each of these measures is expressed, based on the wine absorption spectrum. It should be noticed, however, that these measures are employed in practice only for the assessment of red wines.

Intensity (I) provides an objective quantitative assessment of wine color. The Sudraud method [57] defines wine intensity as the sum of the absorbance measurements at the 420 nm (yellow) and 520 nm (red) wavelengths, respectively,

(13) $$I=A_{420nm}+A_{520nm},$$

where *A_λ_* is the absorbance measurement at wavelength *λ*. Wine intensity in Equation (13) is well correlated to the average *n* of sensory test scores [58], as expressed by

(14) n=23⋅lgI.

Although wine intensity in Equation (13) is a good estimate for the sensorial ranking of wines, as prescribed by Equation (14), it is noteworthy that Equation (14) only holds for red and rose wines, whereas, for white wines, *n* results in negative values and the equation become inapplicable.

In addition to the yellow and red absorption components, the Glories method [59] also considers the absorbance at the 620 nm, i.e., absorption of blue, standing for the absorbance of quinonic forms of free and combined anthocyanins. Accordingly, the intensity expression is redefined as

(15) $$I\prime =A_{420nm}+A_{520nm}+A_{620nm}.$$

Intensity further on enables the assessment of the wine color composition [53,54,55,56], defined by the contribution of each absorption component to the overall intensity, particularly useful in the assessment of wine color saturation, as follows:

(16) $$A_{420nm}[\%]=\frac{A_{420nm}}{I^{\prime }}\cdot 100,$$

(17) $$A_{520nm}[\%]=\frac{A_{520nm}}{I^{\prime }}\cdot 100,$$

(18) $$A_{620nm}[\%]=\frac{A_{620nm}}{I^{\prime }}\cdot 100.$$

Hue (*T*) provides an objective assessment of wine chromaticity and is defined as the angle of the line that connects absorbance components at the 420 nm and 520 nm wavelengths, respectively, on the absorbance spectrum [53], as illustrated in Figure 7b. An estimation of this angle is given by the definition of hue as the ratio of the absorbance measures at the 420 nm and 520 nm wavelengths, respectively [57],

(19) $$T=\frac{A_{420nm}}{A_{520nm}}.$$

With respect to wine aging, red wine assumes a shift from strong red towards orange-red, due to the transition from monomeric to polymeric anthocyanins, respectively. In the spectral domain, this translates to a decrease of *A_520nm_* and an increase of *A_420nm_*, which influences the hue accordingly [60].

Brightness (*dA*[%]) measures the clearness of the wine and is linked to the shape of the absorption spectrum, as prescribed by equation

(20) $$dA[\%]=(1-\frac{A_{420nm}+A_{620nm}}{2\cdot A_{520nm}})\cdot 100,$$

and corresponds to the *A_420nm_*, *A_520nm_* and *A_620nm_* triangle median length, as illustrated in Figure 7b. Brightness measures the contribution of red, i.e., the flavilium cations of the free and combined anthocyanins [53,56], to the wine color. Accordingly, a larger brightness value stands for a dominance of the red color of the wine.

## 3. Results

The laboratory proof of concept of the proposed intra-oral optical sensor is tested in laboratory environment for the intra-oral detection and assessment of wine. Wavelength selective attenuation measures are first assessed in order to have a spectral characterization of the intra-oral optical sensor. Next, measurements on wine samples are carried out. The test results are presented as follows to validate the proposed intra-oral optical sensor.

### 3.1. Spectral Characterization of the Intra-Oral Optic Sensor

**Figure 8:**
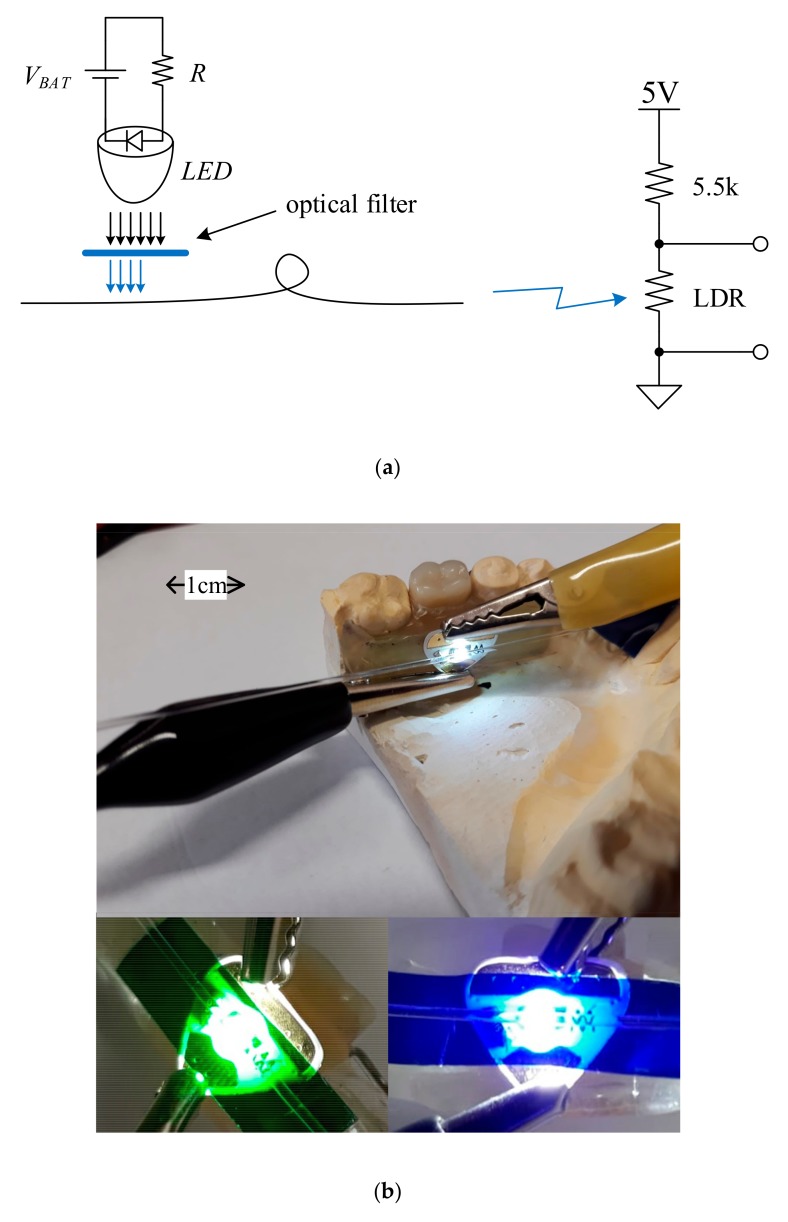
Test setup for the assessment of the point intra-oral sensor spectral attenuation measures: (**a**) schematic diagram, (**b**) practical realization with external LED supply and optical filter foils.

The spectral attenuation of the point intra-oral sensor is expressed in terms of the T-coupler insertion loss, coupling ratio, and excess loss. The test setup is illustrated in Figure 8a.

The optical power from Equations (1)–(3) was replaced with the electrical power measured on a light dependent resistor (LDR). In the measurement setup, the optical fiber output end was focused onto the LDR, connected to a 5 V DC supply in a resistive divider topology with an additional 5.5 K resistance. The output electrical power *P_out,i_*(*λ*), *i* = {1, 2}, was measured at each of the coupler outputs by having each fiber end illuminate the LDR successively. The reference power *P_in_*(*λ*) was measured by having the white LED source (measured broadband spectrum with a 457 nm spectral peak and 8.03 μW emission power) illuminate the LDR over a 1 m long POF optical fiber.

In the absence of a monochromator, as was described in the procedure from [41], wavelength scanning was performed with the use of optical filter foils deployed in between the LED source and the side-emitting fiber, as illustrated in Figure 8b. This allowed for the acquisition of the output radiation power estimate for a discrete set of wavelengths, as listed in Table 1 alongside the spectral attenuation measures. The corresponding spectral attenuation measures were then interconnected with cubic spline interpolation.

**Figure 9:**
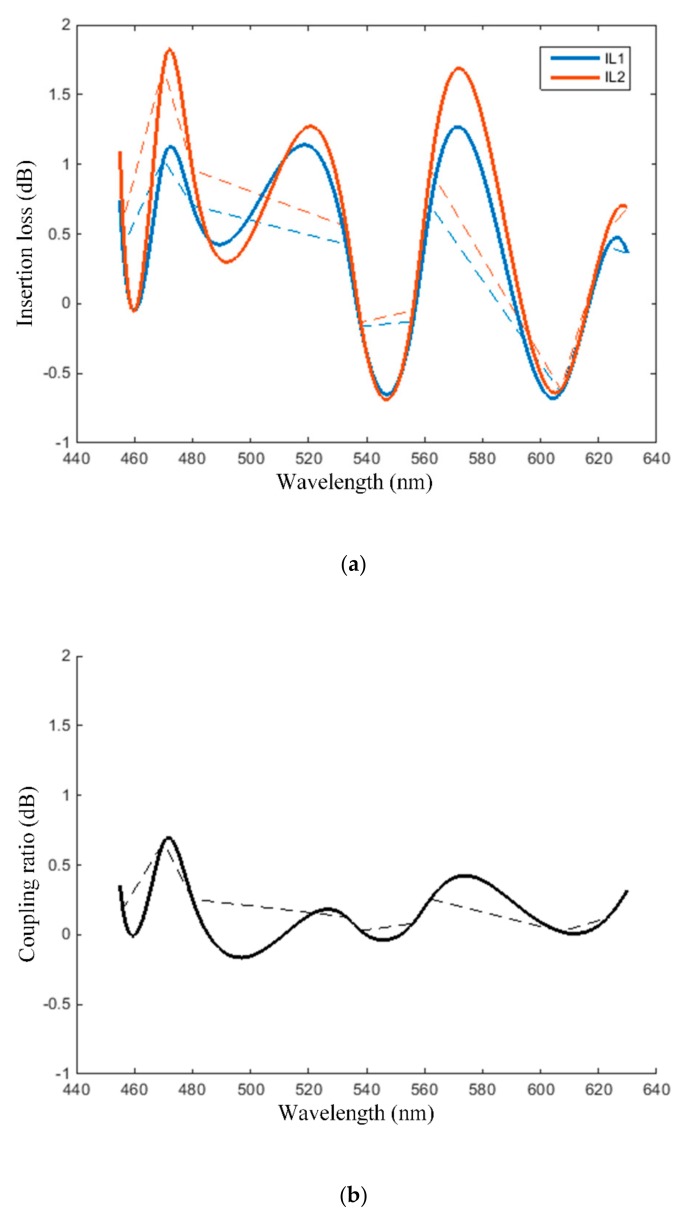

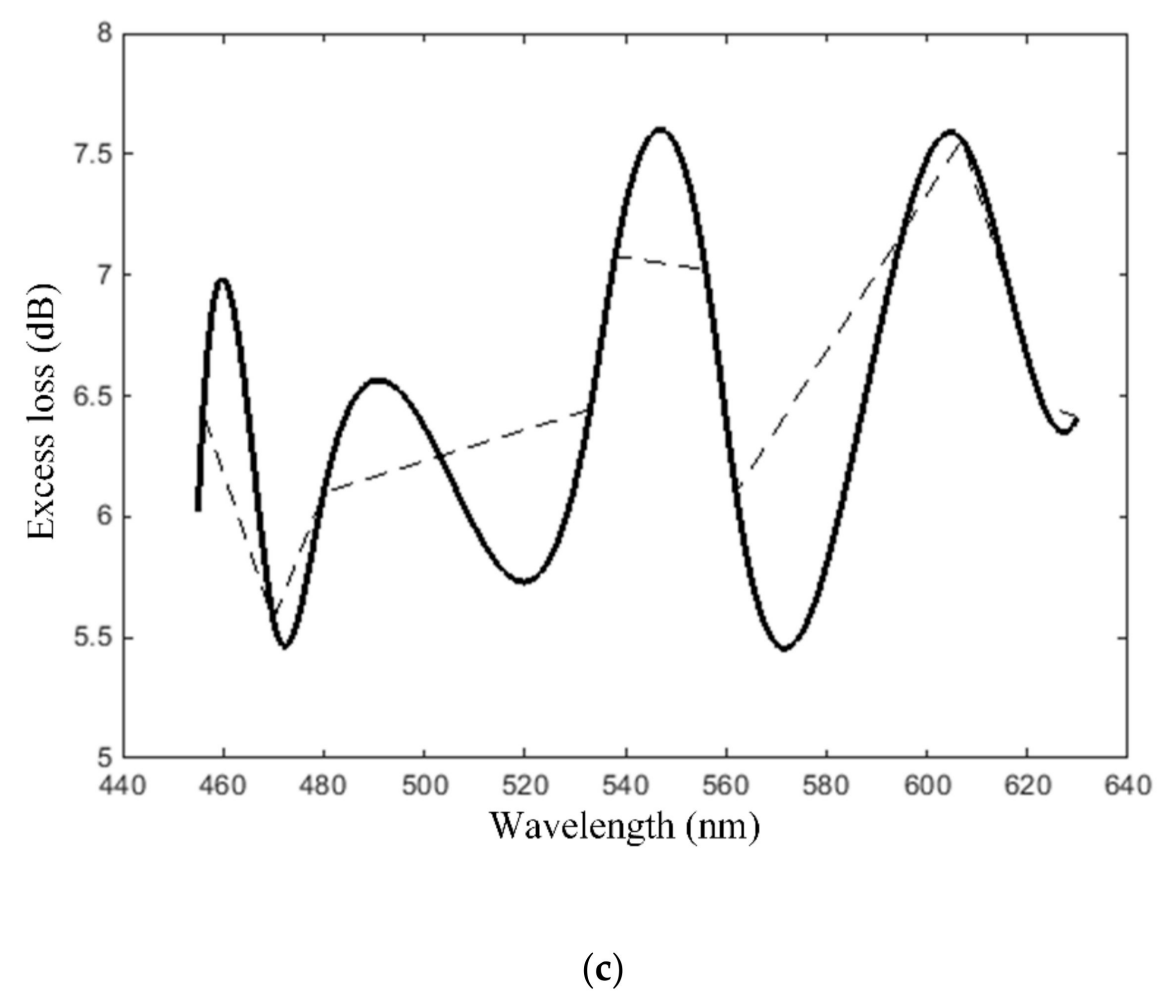
Spectral attenuation measures of the point intra-oral optical sensor expressed in terms of: (**a**) insertion loss, (**b**) coupling ratio, (**c**) excess loss.

The point intra-oral sensor spectral attenuation measures are plotted in Figure 9, with dashed line for 1st order interpolation (straight-line interconnection of the points listed in Table 1) and with continuous lines for cubic spline interpolation.

As illustrated by the insertion loss measures plotted in Figure 9a, as well as by the coupling ratio plotted in Figure 9b, the proposed intra-oral optical sensor can indeed be assimilated to a T coupler with a split ratio close to 50–50%. Indeed, the insertion loss measures at the two fiber ends are approximately equal, and the wavelength dependency of the coupling ratio exhibits a rather small fluctuation—less than 1dB peak-to-peak. Similarly, the excess loss plotted in Figure 9c exhibits a peak to peak variation of maximum 2 dB, and can therefore be assimilated to be relatively flat. Accordingly, the point intra-oral sensor is to be considered transparent with respect to color.

**Figure 10:**
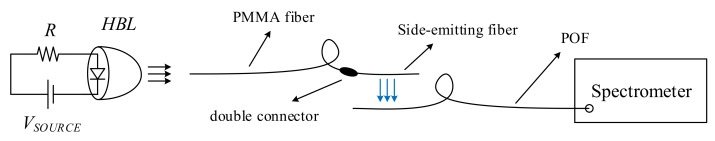
Test setup for the assessment of the wavelength dependent coupling loss of the extended sensing area intra-oral sensor.

A similar procedure was adopted to characterize the spectral behavior of the extended sensing area intra-oral sensor, expressed in terms of wavelength dependent coupling loss. The test setup is illustrated in Figure 10. An addition to the measurement setup is that an external CLM1C-WKW cool white high brightness LED (HBL) (measured broadband spectrum with 445 nm and 567 nm spectral peaks and 174 μW emission power) was used as white radiation source, which applies wideband radiation axially into the side-emitting fiber over a PMMA fiber. This latter PMMA fiber was used as transmission medium to connect the HBL to the intra-oral sensor in order to counter the effect of wavelength selective attenuation of the side-emitting fiber propagated radiation.

**Figure 11:**
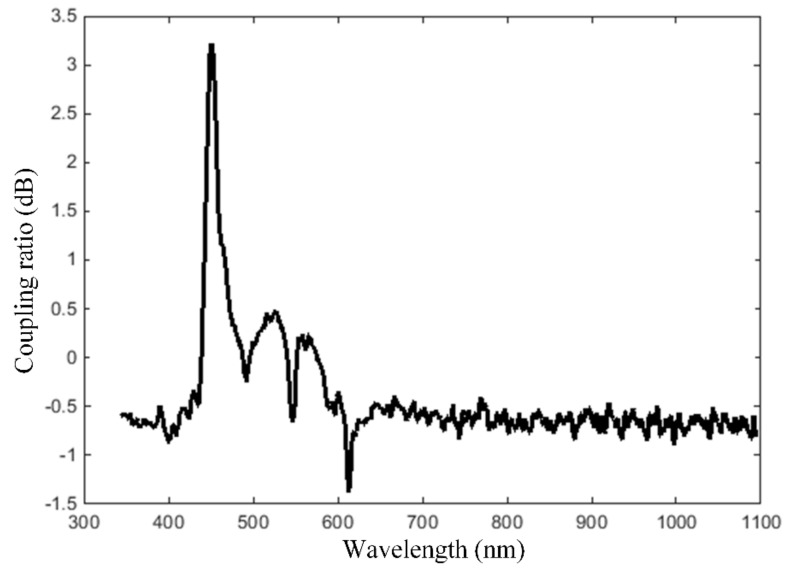
Wavelength dependent coupling loss of the extended sensing area intra-oral sensor.

The wavelength dependent coupling loss of the extended sensing area intra-oral sensor is plotted in Figure 11, illustrating as expected a rather strong loss for short wavelengths, i.e., the blue spectrum. It should be noticed that, besides the superposition of individual loss components, e.g., insertion loss, excess loss, bending loss, and side-emitting fiber input/side-glow relationship which are impractical to be assessed individually for the extended sensing area intra-oral sensor, the wavelength dependent coupling loss measured with the setup from Figure 10 also accounts for the double connector that joins the PMMA fiber to the side-emitting fiber for application of the incident radiation [61].

### 3.2. Wine Color Analysis Results

**Figure 12:**
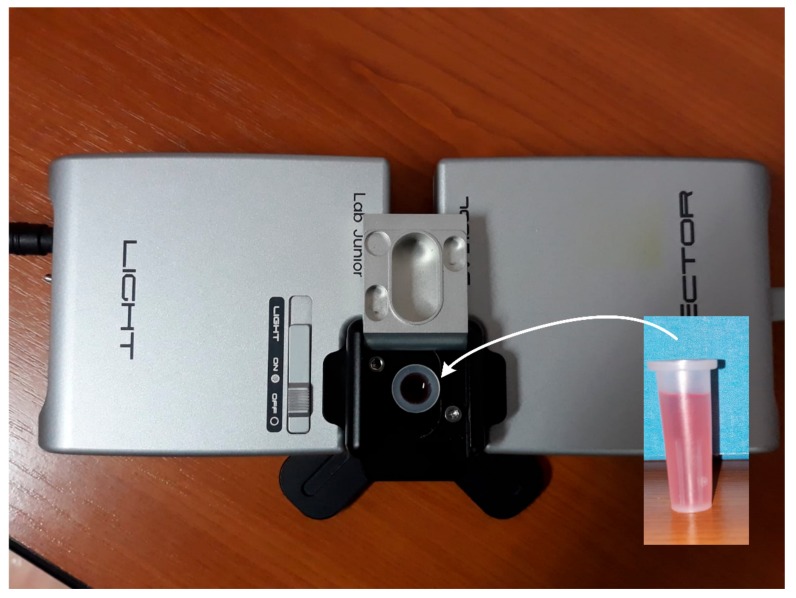
Test setup, consisting of a TH 2100 Tungsten Halogen source and SV 2100 spectrometer, for the acquisition of the reference absorption spectra of the wine samples in plastic cuvettes.

The proposed intra-oral optical sensor implementations were tested to acquire the wine absorption spectrum with eight types of wine: four white wines and four red wines, respectively. A set of reference measurements were first carried out following the standard spectrometric method for wine color determination. The test setup is illustrated in Figure 12 and consists of a KMAC TH 2100 broadband source, i.e., 400–800 nm Tungsten Halogen, and a KMAC SV2100 spectrometer. The samples of wine to be tested for the reference absorption spectra were applied into plastic cuvettes. It should be noticed that plastic cuvettes were employed instead of quartz, which is actually the standard, in order to attenuate the incident radiation on the spectrometer sensor and prevent saturation. The reference absorption spectra will be plotted alongside the wine absorption spectra acquired with the proposed sensors for comparison.

**Figure 13:**
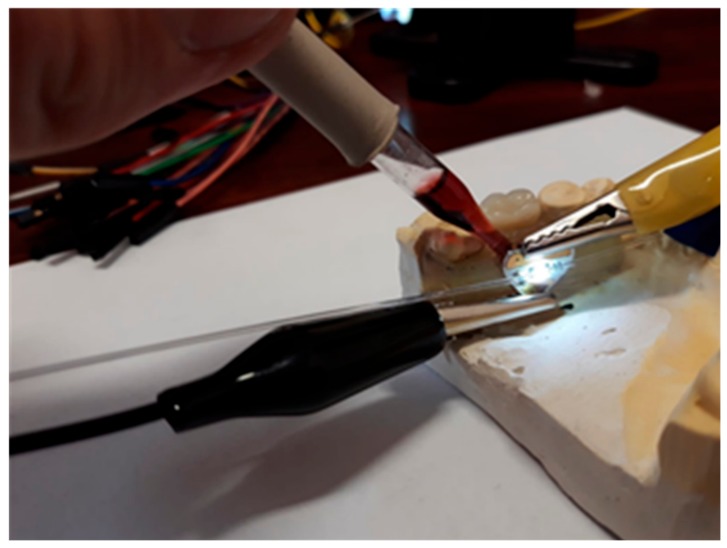
Illustration of the intra-oral optical sensor test procedure for wine color analysis, which assumes having the sensor integrated into a mouthguard and mounted onto a custom jaw mold.

Tests of the proposed intra-oral sensor aim to replicate the intra-oral sensing environment in a wine tasting scenario. The sensor, integrated into a mouthguard and supplied from an external 3 V source, was mounted onto a custom jaw mold as illustrated in Figure 13. Accordingly, the sensing plane is parallel to the lingual slope and perpendicular to the dental arch, thus the analyte will pour through, rather than stagnate, on the sensing surface. The sensor was initially moistened with Ringer solution to replicate saliva in the oral cavity. Wine samples were then applied onto the sensing area with a pipette, and the KMAC SV2100 spectrometer, deployed on the sensor receiving end, acquired the wine spectrum. The sensing area was rinsed with water in between successive tests.

**Figure 14:**
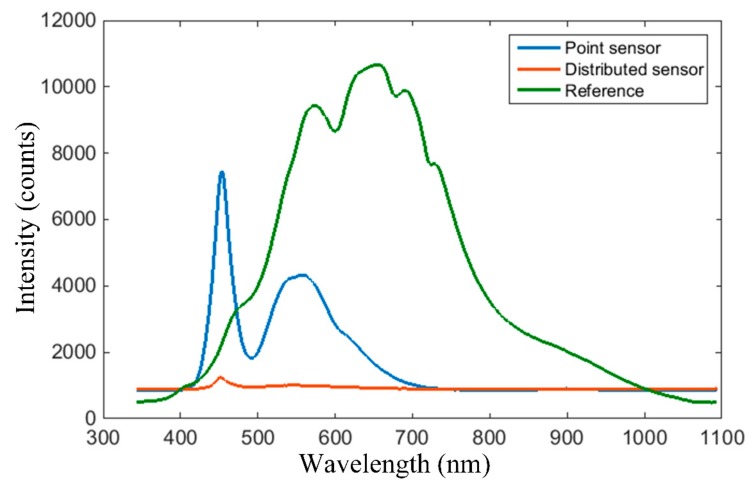
Spectrum of the blank system, i.e., in the absence of the analyte, with a blue line for the point sensor (with white LED source), orange for the extended sensing area sensor (with white LED source), and green line for the reference system (with Tungsten Halogen source).

The spectrum of the blank system, i.e., in the absence of the analyte, is first plotted for reference in Figure 14, to illustrate that spectra acquired in the following tests are indeed a consequence of the analyte, i.e., wine. Further on, our preliminary tests illustrate that water, Ringer solution, and human saliva don’t inflict any modifications to the spectra plotted in Figure 14, and can be considered transparent in the visible range for the proposed sensing systems.

**Figure 15:**
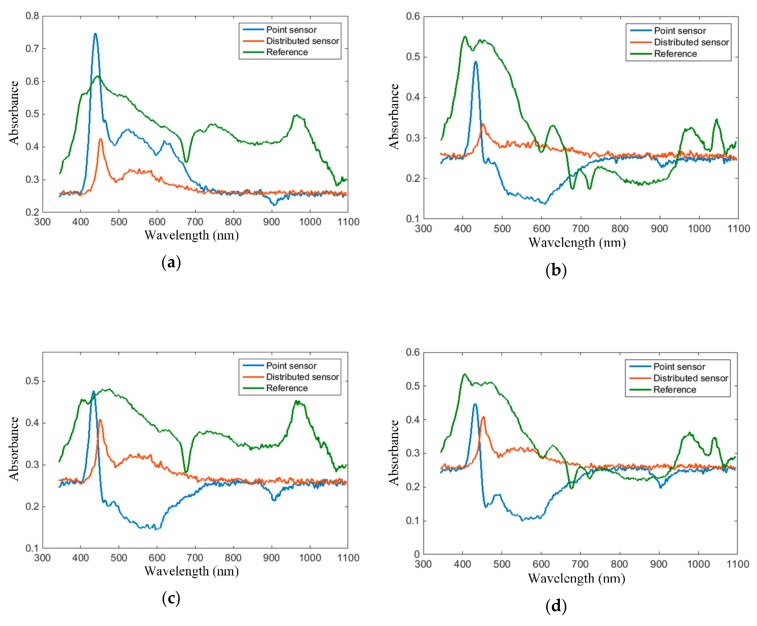
White wine absorption spectra, acquired via the proposed intra-oral sensor implementations, with a blue line for the point sensor, orange line for the extended sensing area sensor, and green line for standard spectroscopic method, respectively: (**a**) blended dry, (**b**) blended medium dry, (**c**) Yellow of Transylvania, (**d**) Sauvignon Blanc.

First, the experimental results obtained with white wine are presented. Four types of white wine have been considered in our tests: Sauvignon Blanc, Yellow of Transylvania (i.e., Fetească Regală, a variety of wine mainly cultivated and produced in Romania), blended dry wine, and blended medium dry wine. The absorption spectra of the white wines considered in our tests, acquired via the proposed intra-oral sensors, are plotted in Figure 15. The results obtained with the point and the extended sensing area sensor respectively are plotted alongside the reference for comparison. 

As illustrated in Figure 15, the white wine absorption spectra follow the specifications of having a spectral peak in the 420–480 nm range, followed by a monotonous roll-off towards longer wavelengths. It should be noticed, however, that the maxima of the absorption spectra acquired with the point and extended sensing area sensors respectively, as listed in Table 2, exhibit a wavelength shift of about 20 nm. This is attributed to the side-emitting fiber wavelength-selective behavior to attenuate short wavelengths inherent to side-emitting fiber operation, this phenomenon being visible on the spectrum of the blank system. Accordingly, wavelengths rejected by the side-emitting fiber don’t even reach being applied onto the analyte to be absorbed by the latter, translating into the 20 nm shift.

It should be noticed that literature doesn’t provide a definition for white wine color characteristics based on the absorption spectra, as is the case for the red wine color characteristics defined in Equations (13)–(19). The CIE Lab color scheme is rather used for the objective assessment of white wine color. Nevertheless, the authors in [62] provide an illustration of the employment of the red wine color characteristics for the assessment of white wines, and, for that purpose, the absorption spectra plotted in Figure 15 allow for the reading of absorption magnitudes at specific wavelengths of interest.

**Figure 16:**
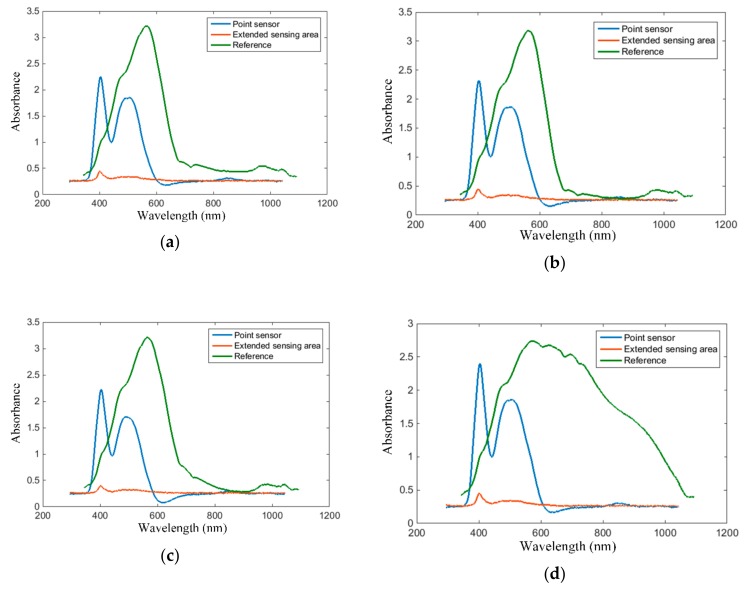
Red wine absorption spectra, acquired via the proposed intra-oral sensor implementations, with the blue line for the point sensor, orange line for the extended sensing area sensor, and green line for standard spectroscopic method, respectively: (**a**) blended medium dry, (**b**) blended medium sweet, (**c**) Fetească Neagră, (**d**) Cabernet Sauvignon.

Second, the experimental results obtained with red wine are presented. Four types of red wine have been considered in our tests: Cabernet Sauvignon, Fetească Neagră (a variety of grapes cultivated in Romania), blended medium dry, and blended medium sweet. The absorption spectra of the red wines considered in our tests, acquired with the proposed intra-oral sensors, are plotted in Figure 16. The results obtained with the point and the extended sensing area sensor, respectively, are plotted alongside the reference for comparison.

As plotted in Figure 16, the red wine absorption spectra follow the specifications of having a peak around 520 nm and a trough around 420 nm. Again, the spectral troughs exhibit a 10–20 nm right shift, as was the case for white wine.

The magnitude of the absorption spectrum at 420 nm, 520 nm and 620 nm, acquired with the point intra-oral sensor, are listed in Table 3 alongside the intensity *I*, estimated sensory test score *n*, modified intensity *I’*, hue *T* and brightness *dA*[%]. To be notices is that the spectral shift is very strong for Fetească Neagră which, according to our measurement results, accounts for a hue value larger than unity.

The magnitude of the absorption wavelength at 420 nm, 520 nm and 620 nm, acquired with the extended sensing area intra-oral sensor, are listed in Table 4 alongside the intensity *I*, estimated sensory test score *n*, modified intensity *I’*, hue *T* and brightness *dA*[%]. Additionally, a multiplicative correction factor of 5 was added to each absorption value to compensate for the strong attenuation inherent to the side-emitting fiber. 

As listed in Table 3 and Table 4, the measurement results are comparable with respect to intensity and estimated sensory test score. The hue measure is also comparable for the blended medium dry and Cabernet Sauvignon wine types. On the other hand, the measures for modified intensity and brightness change due to the *A_620nm_* magnitude which is different for the two sensors. Indeed, *A_620nm_* is considerably smaller than *A_520nm_* and *A_420nm_* for the point sensor, whereas they are comparable for the extended sensing area sensor.

The difference between the results obtained with the point and extended sensing area sensors respectively consists mainly of the magnitude of the absorption spectral components and the 10–20 nm shift in between the spectral peaks. As specified earlier, the spectral shift is attributed to the side-emitting fiber, and, since this phenomenon is known and expected, it can be accounted for during operation. Thus, both sensors are applicable for the intra-oral detection and assessment of wine. Considering however the nature of the application, which assumes real-time assessment of wine intake, a sip of wine would fill the oral cavity and won’t require localizing the presence of wine. From this point of view, we continue our tests with the point sensor.

**Figure 17:**
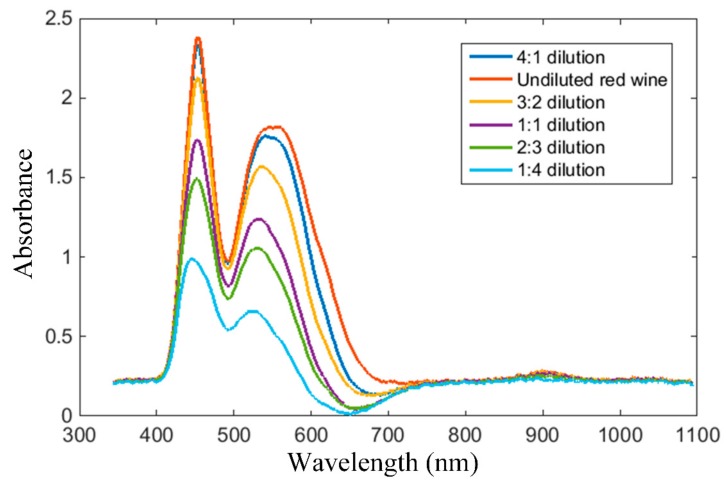
Fetească Neagră red wine absorption spectra, acquired with the point sensor, for different dilutions of wine in saliva.

In the following test scenario, we aimed to detect and assess red wine in the saliva. The normal flow for unstimulated saliva is approximately 0.1 mL/min and for the stimulated saliva is 0.2 mL/min [63]. This amount of saliva does not induce important dilution of the wine; therefore, the wine color is not significantly affected. Rather, saliva contains several peptide families, including the proline-rich proteins that interact with the phenolic compounds in wine, and form complexes that modify the perception of astringency and bitterness [64]. The Fetească Neagră red wine was chosen for this test, as it exhibits the strongest color tonality. Tests were carried out using the point sensor. The absorption spectra for different dilutions of wine in saliva are plotted in Figure 17.

As illustrated, the red wine absorption spectrum follows the specifications depicted in the qualitative plot from Figure 7b. Accordingly, the proposed sensor is applicable for wine identification in the oral cavity. On the other hand, the larger the dilution of wine, the smaller the magnitudes of the absorption spectrum samples. Thus, while it is still possible to detect and assess wine even in cases of rather large dilutions, applicability of the proposed sensor becomes limited for the detection of traces of wine in the saliva.

The optical sensor we developed is also suitable for the assessment of non-alcoholic wines, based on the wine chemistry. The plyphenolic content of wines is responsible for the wine color. Depending on the method used for dealcoholization, the reduced alcohol wines have different physical and chemical properties: higher concentration in polyphenols and organic acids, and increased color intensity [65].

**Figure 18:**
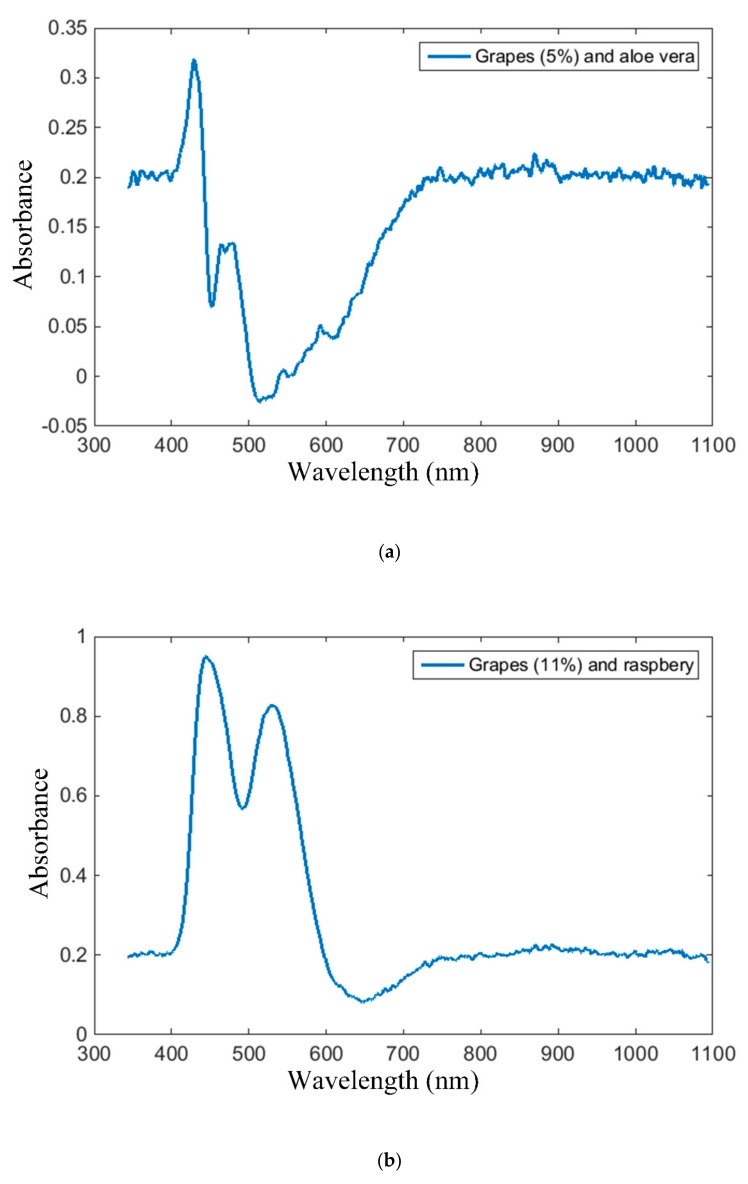

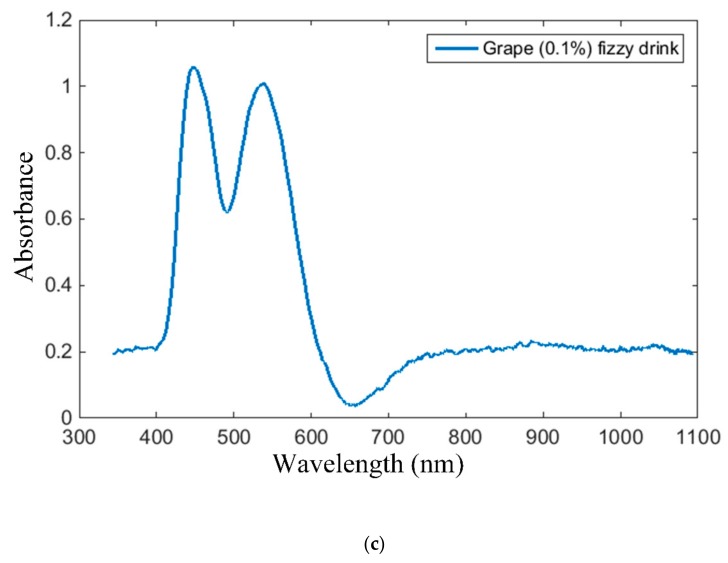
Grape-based drink absorption spectra, acquired via the proposed intra-oral point sensor: (**a**) white grape (5%) and aloe vera, (**b**) white grape (11%) and raspberry, and (**c**) red grape (0.1%) fizzy drink.

With regard to sensor selectivity, we have performed the same spectrometric analyses as for wines on grape-based drinks, using the point sensor. Three types of commercially available drinks have been considered: white grape (grapes 5%) and aloe vera drink, white grape (grapes 11%) and raspberry, red grape fizzy drink (grapes 0.1%). The absorption spectra of the grape-based drinks considered in our tests, acquired with the proposed point sensors, are plotted in Figure 18.

As illustrated, the absorption spectrum of the white grape and aloe vera drink plotted in Figure 18a are clearly distinguished from that of white wines. On the other hand, the shape of the absorption spectra plotted in Figure 18b,c resembles that of the red wines. This is attributed to the red color of the raspberry and the red grape. The magnitudes of the spectral peaks, however, are considerably smaller than those determined for red wines, and, on the other hand, the difference between the two peak magnitudes is considerably smaller than for the red wines. As such, the absorption spectra of the soft drinks is clearly distinguished from the red wine spectra as well.

Another aspect to be considered regards the implementation of the proposed sensors using POFs. Alcohol will dissolve plastic in time and, in the absence of a protective foil, the lifetime of the proposed sensor becomes limited. We have performed five repetitions of each test and have obtained similar results. Then, we have inspected the POFs after each repetition and didn’t find any degradation caused by alcohol.

## 4. Discussion

Test results obtained after testing the proposed intra-oral optical sensors for the detection and assessment of wine in the oral cavity lead to a series of valuable conclusions, enumerated in the discussion which follows.

The wine absorption spectra acquired with the proposed intra-oral sensor and a KMAK SV2100 spectrometer resemble the typical wine absorption spectrum, exhibiting the typical absorption peak, trough and roll-off range. Moreover, the acquired spectra exhibit specific differences with respect to the type of wine being analyzed. Thus, the acquired wine spectra allow for wine discrimination based on the spectral signature.

The wine absorption spectra acquired with the proposed sensor exhibit a resolution fine enough, in both wavelength and amplitude domains, to express the measures of wine colors defined in terms of intensity, chromaticity, and brightness. Accordingly, the proposed intra-oral sensor enables characterization of wine color, which provides valuable objective information with respect to the assessment of wine quality.

As illustrated by all of the test results, the proposed intra-oral optical sensor, with either point or extended area sensing, is validated in the laboratory environment and proves to be applicable for real-time intra-oral sensing.

At this stage, a discussion concerns which of the point or extended sensing area sensor should be chosen for a specific application. Considering the nature of wine testing, which is the scenario targeted in this work, a sip of wine fills the oral cavity. This makes the deployment of an extended sensing area sensor for wine detection unjustified. Moreover, the large attenuation introduced by the side-emitting fiber is too high a price for the target of detecting the same type of analyte all along the dental arch. On the other hand, the target of detecting specific compounds that are uniquely localized inside the oral cavity, e.g., blood in the saliva as was the case in [35], which requires a thorough cartography of the oral cavity, renders the extended sensing area intra-oral sensor well argued.

Further experiments aimed to test the applicability of the proposed sensor for the identification of wine in contact with saliva. Several dilutions of wine with saliva were tested. The acquired absorption spectra follow the typical specifications for wine; therefore, indentification can be performed. The magnitudes of the absorption spectrum samples, however, change with wine dilution, and, although the shape of the spectrum is preserved, assessment of wine must account for this phenomenon.

Sensor selectivity was illustrated with a series of tests carried out with grape-based soft and fizzy drinks. In each situation, the acquired absorption spectrum differs from that of wine. Discrimination of wine from other drinks can thus be performed.

Our tests also illustrate the repeatability of the results. While alcohol clearly alters the parameters of the POF in time, as it dissolves plastic, we were able to confirm that we received similar results for five repetitions of the tests. From this point of view, the 10%–15% alcoholic content of wine won’t limit the sensor lifetime as far as making it inapplicable for in-vivo monitoring.

Furthermore, the proposed sensor performs label-free intra-oral sensing of wine, replicating a real-life sensing scenario. Accordingly, we target the employment of the proposed sensor for in-vivo intra-oral monitoring. It should be noticed that the proposed optical sensor does not detect the alcohol in the wine, but the distinction between the alcoholic and non-alcoholic wines resides in their health effects. Even though the wine consumption could be associated with some health risks due to the alcohol, the polyphenols present in both alcoholic and non-alcoholic wines exert multiple beneficial effects. Moreover, taking into consideration that the alcohol intake is associated with the production of oxidative stress and induces the indirect formation of AGEs, our further research will focus on the inter-relation between alcoholic wine consumption and the salivary levels of AGEs, such as CML.

## 5. Conclusions

This paper presented an intra-oral optical sensor for the intra-oral detection and assessment of wine. The proposed sensor was developed around an optical coupler topology, having an emitter apply lateral illumination onto a D-shaped side-polished plastic optical fiber. Two types of emitters have been employed: a white LED—thus implementing a point sensing, and a side-emitting fiber—thus implementing extended area sensing.

The proposed intra-oral optical sensor resembles the topology of an optical coupler and is characterized in the spectral domain by means of insertion loss, coupling ratio, and excess loss for the point sensor, and wavelength selective coupling loss for the extended sensing area sensor, respectively.

Spectral characterization results illustrate a rather flat variation of the spectral attenuation measures vs. wavelength. Accordingly, the proposed intra-oral sensor is considered to be transparent with respect to color, and is therefore proven to be applicable for spectroscopic sensing applications. However, the proposed sensor illustrates a rather large attenuation on the output/input relationship, and the explanation for this phenomenon is threefold. On one hand, lateral illumination is not focused on the polished surface of the end-emitting optical fiber, thus only a small fraction of the LED emitted light is coupled into the fiber. In addition, having saliva as the optical coupler transmission medium contributes some attenuation. On the other hand, the side-emitting optical fiber from the extended sensing area sensor structure introduces an attenuation inherent to operation. To counter the effect of the attenuation inherently introduced by the side-emitting fiber, the extended sensing area sensor employs a high brightness LED as radiation source.

Based on the spectral characterization results, we have considered the proposed intra-oral optical sensor suitable for the detection and assessment of beverages in the oral cavity, provided they exhibit color. In this respect, we have exemplified the proposed sensor on wine samples, and have tested the proposed sensor for the identification and assessment of wine. To replicate the intra-oral sensing environment in a wine tasting scenario, we have applied wine samples onto the sensing area previously moistened with a Ringer solution. Next, we have tested several dilutions of wine in saliva to demonstrate the applicability of the sensor for intra-oral wine detection and assessment. Finally, selectivity of the proposed sensor was illustrated with an experiment which tests several grape-based drinks.

The proposed intra-oral sensor geometry exploits the advantages of fiber-optics sensing and facilitates integration into a mouthguard. Based on the test results and discussions, the proposed solution holds considerable potential for real-time biomedical applications regarding in-vivo monitoring of food and beverage intake, aiming to forecast the formation of disease-signaling salivary biomarkers. In the context of hybrid patient monitoring, the correlation and combination of data collected by electrochemical sensors and the proposed optical sensor could provide more reliable findings for biochemical and immunological profiling of saliva.

## Figures and Tables

**Table 1:** Resistance values of light dependent resistor measured for different wavelengths of the sensor input, and the corresponding spectral attenuation measures: insertion loss (IL), coupling ration (CR) and excess loss (EL).

Wavelength	*R_LDR_@end_1_*	*R_LDR_@end_2_*	*IL_1_*	*IL_2_*	*CR*	*EL*
455 nm	1.7 kΩ	1.6 kΩ	0.74	1.09	0.35	6.02
456 nm	2.2 kΩ	2 kΩ	0.4	0.6	0.18	6.43
470 nm	1.7 kΩ	1.4 kΩ	1.03	1.7	0.66	5.57
480 nm	1.9 kΩ	1.7 kΩ	0.7	0.95	0.25	6.1
533 nm	2.2 kΩ	2 kΩ	0.42	0.55	0.13	6.44
538 nm	2.9 kΩ	2.8 kΩ	−0.16	−0.13	0.03	7.07
556 nm	2.8 kΩ	2.6 kΩ	−0.12	−0.04	0.08	7.02
562 nm	1.9 kΩ	1.7 kΩ	0.7	0.95	0.25	6.1
607 nm	2.5 kΩ	2.5 kΩ	0.64	−0.61	0.02	7.56
623 nm	2.1 kΩ	2 kΩ	0.4	0.52	0.12	6.46
630 nm	2.3 kΩ	1.9 kΩ	0.35	0.68	0.32	6.41

**Table 2:** White wine absorption peaks, acquired with the point and extended sensing area intra-oral sensors, respectively.

Wine Type	Absorption Peak	
	Point Sensor	Extended Sensing Area Sensor
blended dry	438 nm	452 nm
blended medium dry	432 nm	451 nm
Yellow of Transylvania	434 nm	450 nm
Sauvignon Blanc	431 nm	453 nm

**Table 3:** Red wine absorption spectrum magnitudes and the estimated wine color measures, determined with the point intra-oral sensor.

Wine Type	*A_420nm_*	*A_520nm_*	*A_620nm_*	*I*	*n*	*I’*	*T*	*dA*[%]
blended medium dry	1.5	1.7	0.19	3.2	26.7	3.39	0.88	50.3
blended medium sweet	1.2	1.8	0.17	3	25.2	3.17	0.67	61.9
Fetească Neagră	1.6	1.5	0.07	3.1	26	3.17	n.a.	44.3
Cabernet Sauvignon	1.6	1.8	0.19	3.4	28.1	3.59	0.88	50.2

**Table 4:** Red wine absorption spectrum magnitudes and the estimated wine color measures, determined with the extended sensing area intra-oral sensor.

Wine Type	*A_420nm_*	*A_520nm_*	*A_620nm_*	*I*	*n*	*I’*	*T*	*dA*[%]
blended medium dry	1.5	1.65	1.3	3.15	26.4	4.5	0.9	13.6
blended medium sweet	1.45	1.7	1.3	3.15	26.4	4.5	0.85	17.6
Fetească Neagră	1.45	1.55	1.3	3	25.2	4.35	0.93	9.6
Cabernet Sauvignon	1.5	1.65	1.4	3.15	26.4	4.55	0.9	12.1
